# IMMERSIVE VISUALIZATION OF MOVEMENT IN PATIENTS WITH HAEMOPHILIC ANKLE ARTHROPATHY: MULTICENTER, SINGLE-BLIND, RANDOMIZED CLINICAL TRIAL

**DOI:** 10.2340/jrm.v56.40775

**Published:** 2024-09-30

**Authors:** Roberto UCERO-LOZANO, Raúl PÉREZ-LLANES, Rubén CUESTA-BARRIUSO, Elena DONOSO-ÚBEDA

**Affiliations:** 1 Department of Physiotherapy, European University of Madrid, Madrid; 2 InHeFis Research Group, Instituto Asturiano de Investigación Sanitaria (ISPA), Oviedo; 3 Department of Physiotherapy, University of Murcia, Murcia; 4 Department of Surgery and Medical-Surgical Specialties, University of Oviedo, Oviedo; 5 Department of Physiotherapy, Catholic University San Antonio-UCAM, Murcia, Spain

**Keywords:** haemophilia, virtual reality exposure therapy, joint pain, conditioned pain modulation, pressure pain threshold, range of motion

## Abstract

**Objective:**

To evaluate the efficacy of immersive movement observation in adult patients with haemophilic ankle arthropathy.

**Design:**

Multicentre, single-blind, randomized clinical trial.

**Subjects:**

48 patients with haemophilia.

**Methods:**

Patients were randomly allocated to 2 groups (180º immersive video-based visualization of movement and a control group with no intervention). Twenty-eight consecutive 15-min home sessions, 1 per day, of immersive visualization of ankle flexion–extension movement were carried out. Three evaluations were performed: pretreatment (T0), post-intervention (T1), and at 16 weeks’ follow-up (T2). The primary variable was joint-pain intensity (visual analogue scale). The secondary variables were conditioned pain modulation (Conditioned Pain Modulation Index), pressure pain threshold (pressure algometer), range of motion (goniometry) and kinesiophobia (Tampa Scale of Kinesiophophia).

**Results:**

There were intergroup differences in pain intensity (F = 37.14; *p* < 0.001), conditioned pain modulation (F = 5.40; *p* = 0.006), and dorsal (F = 19.17; *p* < 0.001) and plantar (F = 9.27; *p*<0.001) ankle flexion. More than 50% of experimental group patients exhibited changes exceeding the minimum detectable change in pain intensity (MDC = 0.43), and the pressure pain threshold in the extensor carpi radialis longus muscle (MDC = 1.34) and malleolus (MDC = 4.93).

**Conclusions:**

180º immersive video-based visualization of movement can improve the intensity of pain, conditioned pain modulation, and ankle range of motion in patients with haemophilic ankle arthropathy.

## LAY ABSTRACT

Patients with haemophilia have a congenital coagulation problem. The most severe cases can have spontaneous bleeding that affects muscles and joints. Many of them suffer joint degeneration (haemophilic arthropathy) and chronic pain. Pain occurs in the brain like an alarm response and provokes cerebral changes. Indeed, this is the reason why we can use a virtual reality approach to treating pain. In this study we compare the effects of adding 15 min of seeing a movement wearing virtual reality googles in comparison with continuation of patients’ life for 28 days in patients with ankle haemophilic arthropathy. We evaluated before and after the 28 days, and 16 weeks after finishing the treatment. The results of this study show that this treatment can improve the intensity of pain perceived by patients and their ankle mobility in patients with haemophilic ankle arthropathy.

Haemophilia is a chronic, congenital, and rare disease (1). Patients with haemophilia suffer from bleeding due to a deficiency of one of the clotting factors. The most common types of haemophilia are: haemophilia A (factor VIII deficiency) and haemophilia B (factor IX deficiency) (2). The severity of the disease is classified according to plasma levels of clotting factor: severe (<1%), moderate (1–5%), and mild (5–40%) (3).

Joint bleeding is characteristic of this disease (4). Such bleeding events can occur with minimal trauma (5). The joints most often affected are ankles, knees, and elbows (2). The recurrence of haemarthrosis causes changes in the synovial membrane that induce joint degeneration (haemophilic arthropathy) (5). The most disabling clinical features of this arthropathy include chronic pain and decreased joint range of motion (ROM), progressively leading patients to reduce their activity and social participation (4).

Pain is defined as “an unpleasant sensory and emotional experience associated with, or resembling that associated with, actual or potential tissue damage” (6), beyond mere nociception. Acute pain is considered a failure of avoidance behaviour, transforming nociceptive activity into conscious pain. However, chronic pain lacks this physiological protective function (7).

Unhelpful beliefs concerning pain have developed, perceiving the body as something fragile and vulnerable, associating the experience of pain with structural damage. This evaluative bias perpetuates beliefs and fear of pain (8). This fear may seem useful to modify behaviours after an injury. However, it can be a source of long-term disability, if learning and exposure strategies are not offered to cope with fear. Learning can help the patient gain control over this process (8). Chronic pain of moderate intensity with peaks of severe pain is highly prevalent in patients with haemophilia. This pain is correlated with the degree of kinesiophobia and catastrophism (9).

Motion visualization consists of watching a video or live actions performed by an actor (10). The patient, through the activation of mirror neurons, transforms the visual input into activation of the same cortical areas that allow motor execution in real life (10, 11). The therapeutic use of virtual reality (VR) has shown its effectiveness in improving the range of motion and functionality in patients with total knee and hip arthroplasty (10).

The aim of this study was to evaluate the effectiveness of a movement visualization intervention using a 180-degree immersive video in first-person perspective in patients with haemophilic ankle arthropathy.

## METHODS

### Study design

A multicentre, single-blind, randomized clinical study was undertaken.

### Patient recruitment and selection

The study was carried out between November 2021 and February 2022 in 6 regions of Spain (Andalusia, Aragon, Castilla y León, Galicia, Madrid, and Murcia).

The inclusion criteria in the study were: (*i*) over 18 years of age; (*ii*) medical diagnosis of haemophilia A or B; (*iii*) severe haemophilia phenotype (<1% of FVIII/FIX); (*iv*) bilateral ankle arthropathy; and (*v*) more than 5 points on the Haemophilia Joint Health Score (12) of joint damage.

Patients who: (*i*) did not present ankle pain; (*ii*) with cognitive impairments; (*iii*) diagnosed with epilepsy or with severe vision problems; (*iv*) receiving physiotherapy treatment during the study; and (*v*) who did not sign the informed consent were excluded from the study.

All patients received regular haemostatic monitoring at their referral hospital. The therapeutic regimen of the patients prescribed by their haematologist was not modified.

### Ethical considerations

This study was conducted in accordance with the Declaration of Helsinki. The subjects were informed of the risks and benefits of the study. All subjects signed the informed consent document. The project was approved by the CEIm of the Virgen de la Arrixaca University Hospital (ID: 2020-2-9-HCUVA). The research project was registered prior to the study (www.clinicaltrials.gov; ID: NCT04549402).

### Measurement instruments

The primary variable was intensity of joint pain evaluated with the visual analogue scale (13). This scale has shown moderate reliability (14) in patients with chronic musculoskeletal pain. It assesses the intensity of the pain perceived by the patient on a 10-cm line. Within a range from 0 to 10, 10 indicates “the worst pain”.

The secondary variables were conditioned pain modulation, pressure pain threshold, ankle range of motion, and kinesio-phobia.

Conditioned pain modulation was measured using the Conditioned Pain Modulation Index (CPMI) (15). This procedure evaluates diffuse descending pain modulation by facilitating or inhibiting responses to a conditioned stimulus (15), with moderate reliability (16). For the evaluation, tonic pain was caused by pressing on a non-painful area (15). The threshold was first measured at the base of the dorsal part of the distal phalanx of the thumb and subsequently the conditioned stimulus was triggered using the ischaemia test (16) in the contralateral upper limb; the pain was rated on a numerical pain scale (17). The measurement was then repeated. The results were transformed according to the CPMI score. A positive result indicates pain inhibition and activation of the CPM phenomenon (18).

With a pressure gauge (model Wagner FDIX, Wagner Instruments, Riverside, CT, USA) (19) the pressure pain threshold was measured. Pressure was progressively applied until the feeling began to be painful (20). The pressure pain threshold was measured, bilaterally, in the ventral region to the lateral malleolus (21), in the same metamere (5th lumbar vertebra), and in an area with no direct neurological relationship (extensor carpi radialis longus) (19).

Using an analogue goniometer, the range of motion was measured. This instrument has shown excellent reliability (22). The joint range in plantar and dorsal flexion was measured (23). The unit of measurement is the degree (the higher the degree, the greater the mobility).

With the Spanish version of the Tampa Scale of Kinesiophophia questionnaire (TSK-11SV) (24) fear of movement was evaluated. This instrument offers moderate reliability (24). The scoring range is 11–44 points (a higher score indicates greater kinesiophobia).

Table I indicates the main characteristics of the measuring instruments used in the study.

**Table I:** Measuring instruments used in the evaluation of dependent variables

Measurement tool	Variable	How to use	Reliability	Units (range)
Visual analogue scale	Pain intensity	10-cm line. Patients must mark on the line their usual average joint pain intensity during the last week 0 “no pain”, 10 “the worst imaginable pain”	0.60–0.77†	Score(0–10)
Conditioned Pain Modulation Index	Conditioned pain modulation	PPT_pre_: PPT at the base of the dorsal 2^nd^ phalanx of the thumb Ischsemic conditioned pain stimulation on the contralateral upper limb. A sphygmomanometer was placed on the arm 14 cm from the ulnar fossa. It was inflated to 240 mmHg. Patients should lift a weight of 2 kg up to a maximum of 45 repetitions, or until they indicated pain greater than 7/10 on the NRS PPT_post_: New PPT measure at the base of the dorsal 2nd phalanx of the thumb	0.60–0.75†	Score
Pressure algometer	Pressure pain threshold	Pressure was applied to the chosen point and increased at a rate of approximately 50kPa/s until the patient signalled a painful sensation	0.82–0.97†	*n*/cm^2^
Analogic goniometer	Range of motion	Measured in plantar and dorsal flexion with patient in supine position Lateral malleolus was used as the axis of movement, the fibular diaphysis as the fixed arm and the 5th metatarsal as the mobile arm	0.85–0.96†	Degrees(0–45)
Tampa Scale of Kinesiophobia	Kinesiophobia	Self-reported. 11 Items that rated from 1 (strongly disagree) to 4 (strongly agree)	0.79^α^	Score(11–44)

† ICC: intraclass correlation coefficient;

α Cronbach’s alpha. PPT: pressure pain threshold; NRS: numeric rating scale.

In the pre-treatment evaluation, the anthropometric, sociodemographic, and clinical variables were evaluated. Joint condition was measured using the Haemophilia Joint Health Score (12). This scale, specific for patients with haemophilia, evaluates 8 items: inflammation and duration of inflammation, pain, atrophy and muscle strength, crepitus, and loss of flexion and extension. Each joint, knees, ankles, and elbows, is scored from 0 to 20 points (maximum joint damage).

Prior to recruitment, intra- and interobserver reliability was calculated based on 7 patients with ankle arthropathy. High intraobserver reliability was noted in pain intensity (ICC = 0.98), pressure pain threshold in the malleolus (ICC = 0.89) and extensor carpi radialis longus (ICC = 0.97), and plantar (ICC = 0.90) and dorsal ICC = 0.86) flexion. Intraobserver reliability was moderate in the variables for conditioned modulation (ICC = 0.76) and pressure pain threshold at L5 (ICC = 0.77).

### Randomization

Randomization was performed with a computerized randomization procedure using permuted blocks of 8 subjects in each of the 6 recruitment centres. In each block, the 2 allocation possibilities (experimental or control) were randomly coded with 8 sequence alternatives. This process was carried out by an assistant blinded to group assignment and patient identification.

### Intervention

**Fig. 1:**
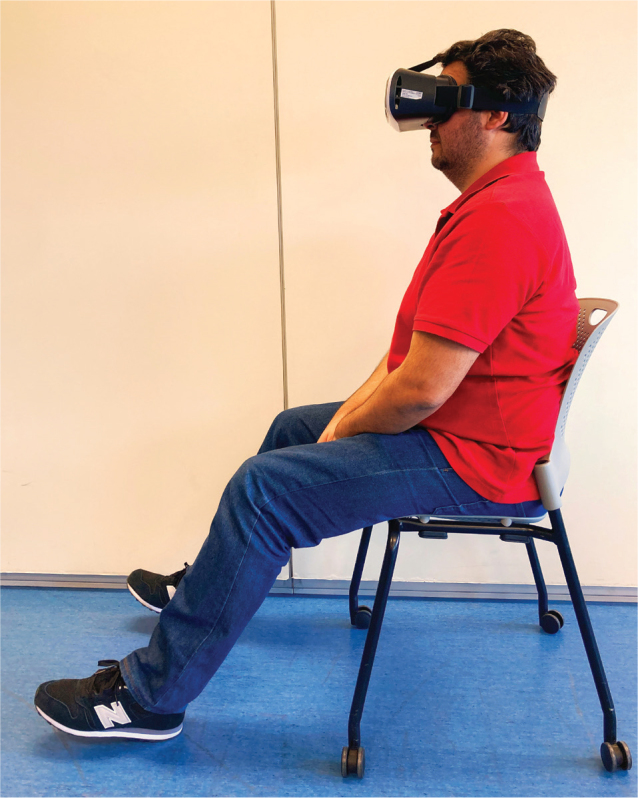
Patient performing the intervention of 180-degree immersive VR motion visualization.

An intervention of immersive visualization of ankle flexion–extension movement was carried out in patients included in the experimental group. A first-person-perspective 180-degree immersive video was used. This video was shown on the patient’s smartphone. The operating system was not an excluding factor. For immersive visualization, the smartphone was housed in virtual reality glasses (3D virtual reality glasses with remote control; model Qmax; https://www.qmax.bg/product-category/phones-and-tablets/gadgets-smart-technology/3d-glasses/) (25). The video was hosted on a YouTube channel^®^ with access from the He-Mirror App^®^, designed *ad hoc* for the study. The subjects sat in a chair, with their feet relaxed and resting only on their heels. The intervention consisted of 28 consecutive home sessions, 1 per day. Each session lasted 15 min. During the session the patient needed only to watch the movement of both ankles in the video, without imagining the movement or performing it. Fig. 1 shows the performance of the intervention by 1 of the patients included in the study.

Patients included in the control group did not perform any visualization intervention, continuing with their usual routine of daily life activities and haemostatic control.

### Procedure

The patients were evaluated at 3 time points: at baseline (T0), after the intervention (T1), and after 16 weeks’ follow-up (T2). All measurements were performed under the same conditions reproducing the same protocol. All the evaluations were carried out by the same rater, blinded to subject allocation to the study groups. The rater, with more than 20 years of clinical experience, was trained in the evaluation of patients with haemophilia. In the first measurement, the anthropometric and clinical variables were assessed.

### Sample size

The sample size was calculated using the statistical package G*Power (version 3.1.9.2; Heinrich-Heine-Universität Düsseldorf, Germany). Assuming a high effect size (d = 0.81) (26), with an alpha level (type I error) of 0.01 and a statistical power of 90% (1-β = 0.90), a sample size of 20 patients in each group was estimated. Accounting for potential dropouts during the experimental phase and the follow-up period, an additional 20% were recruited. Thus, 48 patients with haemophilia and ankle arthropathy were included in the study.

Fifty-five patients with haemophilia were invited. Four failed to meet the selection criteria and 1 patient was excluded because of scheduled orthopaedic surgery. Two patients declined to participate.

### Statistical analysis

Data were analysed using SPSS 19.0 software (IBM SPSS Statistics for Windows; IBM Corp, Armonk, NY, USA). According to *a priori* sample size calculation parameters, the statistical significance was set at *p* < 0.01 for a 99% confidence interval (CI). Full analysis set according to ICH guidelines was carried out.

The intra- and inter-rater reliability analysis was performed with the two-way random intraclass correlation coefficient. Although some data were not normally distributed, F-tests are robust in terms of type I error, and regardless of the manipulated conditions are considered a valid option for non-parametric distributions (27). The intergroup effect was calculated with repeated measures ANOVA. The Greenhouse–Geisser correction was considered when Mauchly’s test rejected sphericity (28). The effect size of the F-tests was analysed using the eta-squared coefficients (η^2^) and was interpreted as a small (η^2^ = 0.01), medium (η^2^ = 0.06), and large (η^2^ = 0.14) effect size (29).

The minimum detectable change (MDC) was obtained by estimating the standard error of measurement (SEM) with the formula: SEM = SD_pre_*√1-ICC (30). The formula (MDC = Z-score*√2*SEM) was used to obtain the MDC. The confidence level was set at 95% (Z-score = 1.96) (31). Finally, the proportion of patients whose change after the intervention exceeded the value indicated by the MDC was calculated.

## RESULTS

**Fig. 2:**
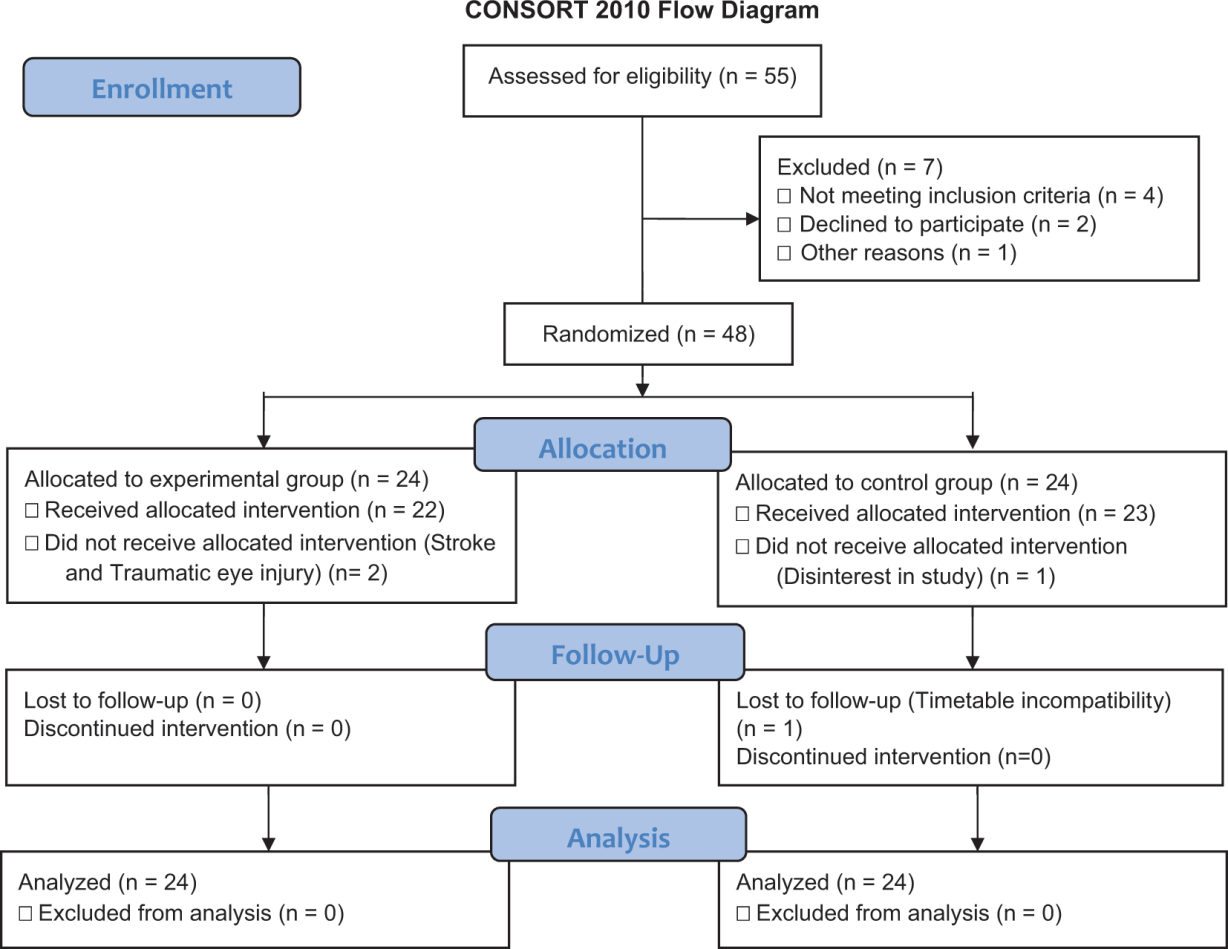
Flowchart of randomization and follow-up.

Forty-eight patients were included in the study. During the experimental phase, 3 patients dropped out of the study. Another patient did not participate in the follow-up evaluation due to timetable incompatibility. Fig. 2 shows the flow diagram of the study.

The mean age of the patients was 39.85 (SD 6.89) years. The majority of patients had a diagnosis of haemophilia A (85.4%) and were receiving prophylactic treatment (64.5%). Four patients (20.8%) had developed inhibitors. None of the patients developed ankle hemarthrosis during the experimental phase. Table II lists the descriptive characteristics of the patients.

**Table II:** Descriptive statistics of patients included in the study, depending on the group

Variables	Experimental group (*n* = 24)	Control group (*n* = 24)
Age, years, mean (SD)	40.58 (8.26)	39.12 (5.20)
Weight, kg, mean (SD)	80.31 (10.74)	84.20 (4.53)
Height, cm, mean (SD)	173.17 (7.17)	177.21 (4.75)
Ankle joint damage (0–20), mean (SD)	11.98 (2.78)	11.33 (2.91)
Type of haemophilia, *n* (%)		
A	20 (83.3)	21 (87.5)
B	4 (16.7)	3 (12.5)
Treatment, *n* (%)		
On-demand	15 (62.5)	16 (66.7)
Prophylaxis	9 (37.5)	8 (33.3)
Inhibitors, *n* (%)		
Yes	6 (25)	4 (16.7)
No	18 (75)	20 (83.3)
Drugs, *n* (%)		
No	5 (20.8)	4 (16.7)
Paracetamol	8 (33.3)	9 (37.5)
NSAIDS	2 (8.4)	3 (12.5)
COX-2 inhibitors	9 (37.5)	8 (33.3)

SD: standard deviation; NSAIDS: nonsteroidal anti-inflammatory drugs.

### Pain intensity

When analysing the intergroup effect we observed statistically significant differences (F[1.74;162.67] = 37.14). In the pairwise comparison analysis there were significant changes in T1–T0 (99% CI (–0.75; –0.27) and T2–T0 (99% CI –0.81; –0.15). The value of the minimum detectable change was 0.43 points. Changes greater than the MDC after the intervention were reported in 75% of experimental group patients.

### Repeated measures analysis

There were statistically significant intergroup differences (*p* < 0.001) in conditioned pain modulation (F[2;92] = 5.40). There were also statistically significant differences in dorsal (F[1.76;165.98] = 19.17) and plantar (F[2;188] = 9.27) flexion. However, there were no statistically significant differences (*p* > 0.01) in the pressure pain threshold in the extensor carpi radialis longus (F[2;92] = 3.25), malleolus (F[2;92] = 4.11), or fifth lumbar vertebra (F[1.54;71.17] = 4.62), or in kinesiophobia (F[2;92] = 2.92). Table III gives the results of the repeated measures analysis.

**Table III:** Mean (and 99% confidence interval) and intergroup analysis of study

Variables	Assessment	Experimental Mean (99%CI)	Control group Mean (99%CI)	Interaction time*group
Joint pain(0–10)	Baseline	3.57 (2.80; 4.43)	3.22 (2.33; 4.12)	F = 37.14; ***p* < 0.001**η^2^ = 0.28
	Post-treatment	2.34 (1.55; 3.24)	3.43 (2.49; 4.33)	
	Follow-up	2.26 (1.57; 2.97)	3.58 (2.69; 4.48)	
Conditioned Pain Modulation Index (score)	Baseline	0.18 (0.10; 0.27)	0.36 (0.25; 0.49)	F = 5.40; ***p* = 0.006**η^2^ = 0.10
	Post-treatment	0.16 (0.07; 0.25)	0.34 (0.19; 0.49)	
	Follow-up	0.27 (0.13; 0.41)	0.28 (0.12; 0.46)	
ECRL Pressure pain threshold (N)	Baseline	37.80 (28.81; 46.31)	39.97 (32.25; 49.77)	F = 3.25; *p* = 0.04η^2^ = 0.66
	Post-treatment	42.62 (33.70; 51.56)	41.88 (34.13; 51.07)	
	Follow-up	42.48 (33.30; 52.46)	45.47 (37.54; 54.48)	
Malleolus pressure pain threshold (N)	Baseline	56.32 (43.83; 70.95)	58.49 (46.84; 71.30)	F = 4.11; *p* = 0.02η^2^ = 0.08
	Post-treatment	64.39 (50.10; 79.68)	59.68 (47.27; 72.43)	
	Follow-up	65.16 (52.61; 83.22)	63.28 (50.32; 76.15)	
5L pressure pain threshold (N)	Baseline	56.76 (45.89; 67.85)	57.92 (46.71; 69.83)	F = 4.62; *p* = 0.02η^2^ = 0.09
	Post-treatment	66.76 (51.74; 82.49)	58.56 (46.26; 70.66)	
	Follow-up	66.66 (51.37; 82.92)	59.19 (45.82; 71.40)	
Dorsal flexion(degree)	Baseline	–2.50 (–5.57; 0.27)	0.85 (–0.82; 2.52)	F = 19.17; ***p* < 0.001**η^2^ = 0.16
	Post-treatment	–0.85 (–3.54; 1.77)	0.53 (–1.21; 2.27)	
	Follow-up	–0.94 (–3.40; 1.62)	0.13 (–1.59; 1.85)	
Plantar flexion(degree)	Baseline	37.48 (33.76; 41.72)	35.23 (32.26; 38.10)	F = 9.27; ***p* < 0.001**η^2^ = 0.09
	Post-treatment	38.65 (34.48; 43.55	34.77 (31.62; 37.66)	
	Follow-up	38.13 (34.18; 42.90)	34.19 (30.67; 37.27)	
Kinesiophobia(11–44)	Baseline	26.29 (22.60; 30.29)	26.67 (23.82; 29.50)	F = 2.92; *p* = 0.06η^2^ = 0.06
	Post-treatment	24.04 (21.39; 27.00)	26.96 (23.87; 29.92)	
	Follow-up	24.37 (21.30; 27.80)	27.00 (24.00; 30.05)	

ECRL: extensor carpi radialis longus; 5L: 5th lumbar vertebra; 99% CI: 99% confidence interval; Sig.: significance; η^2^: eta-square; N: Newtons.

*p* < 0.01 with a 99% confidence interval (shown in bold) was considered statistically significant.

### Minimum detectable change

When calculating the value of the minimum detectable change (MDC = 0.64), 37.5% of the patients in the experimental group showed equal or greater changes after the intervention in conditioned pain modulation. More than half of the patients who underwent the intervention showed values higher than the MDC in the pressure pain threshold of the extensor carpi radialis longus (MDC = 1.34) and malleolus (MDC = 4.93). More than 20% of the patients showed greater changes in dorsal (MDC = 3.51) and plantar (MDC = 2.91) flexion. When calculating the MDC of kinesiophobia, through the previously calculated SEM (32), we noted how 25% of the patients exhibited changes greater than the MDC (4.41). The results of the calculation of the minimum detectable change are respectively listed in Table IV.

**Table IV:** Calculation of the minimum detectable change in the secondary study variables

Variables	ICC_2,1_ (SD_dif_)	SEM	MDC	MDC_p_
Joint pain	0.98 (0.15)	0.02	0.43	Experimental group: 75%
				Control group: 0%
Conditioned Pain Modulation Index	0.76 (0.11)	0.05	0.64	Experimental group: 37.5%
				Control group: 0%
ECRL pressure pain threshold	0.97 (1.36)	0.24	1.34	Experimental group: 58.3%
				Control group: 0%
Malleolus pressure pain threshold	0.89 (9.57)	3.17	4.93	Experimental group: 54.17%
				Control group: 0%
5L pressure pain threshold	0.77 (7.59)	3.64	5.28	Experimental group: 41.66%
				Control group: 0%
Dorsal flexion	0.82 (3.78)	1.60	3.51	Experimental group: 20.83%
				Control group: 0%
Plantar flexion	0.88 (3.19)	1.11	2.91	Experimental group: 25%
				Control group: 0%
Kinesiophobia	–	2.54	4.41	Experimental group: 25%
				Control group: 0%

ECRL: extensor carpi radialis longus; 5L: 5th lumbar vertebra; ICC_2__,1_: two-way random intraclass correlation coefficient; SD_dif_: standard error of the difference in the intra-rater evaluation; SEM: standard error of measurement; MDC: minimum detectable change; MDCp: proportion of minimal detectable change.

## DISCUSSION

The aim of this study was to evaluate the effectiveness of an intervention of immersive visualization of movement in patients with haemophilic ankle arthropathy. After the intervention there were intergroup changes in pain intensity, conditioned pain modulation, and range of motion. We noted a relevant clinical effect on the variables pain intensity and pressure pain threshold.

In patients with musculoskeletal pain, there is a relationship between motor deficits and pain, which may precede pain, being associated with cortical reorganization and changes in cortical processing (33). This cortical reorganization occurs in the primary somatosensory and motor cortex. This phenomenon has been proposed as the reason for the inconsistency between motor intention and sensory feedback that may persist, causing the affective experience of pain (33). Chronic perception of joint pain in patients with haemophilic arthropathy is common (4).

Exposure to virtual reality can improve the intensity of pain in patients with knee arthroplasty (34). While pain and disuse can cause neuroplastic changes that lead to cortical reorganization, visualization of movement promotes neuroplastic and motor control changes (10). The persistence of pain over time causes neuroplastic changes in patients with haemophilia. Maintained changes in pain after the follow-up period in the experimental group patients may be due to the influence on the previous painful experience.

Statistically significant changes do not necessarily reflect a clinically significant change. Calculation of the minimum detectable change (MDC) offers the minimum change in the score that, with 95% confidence, reflects a real clinical change and not one due to a measurement error (35). More than 50% of the patients included in the experimental group exhibited changes greater than the MDC in intensity of pain. These high percentages demonstrate how the results are able to detect the true clinical change from this intervention in patients with haemophilia. According to such values, the observed changes were homogeneous in a significant percentage of patients, thus suggesting the effectiveness of this intervention in patients with haemophilic ankle arthropathy.

Conditioned pain modulation is the joint effect of endogenous pathways that enhance or reduce the effects of afferent noxious stimuli (36), being an endogenous phenomenon of pain inhibition. Conditioned pain modulation is significantly impaired in a large number of patients with chronic pain (37). The reported changes may be due to the fact that, like other patients with chronic pain, haemophilia patients suffer from such a deterioration of this modulation system. Visualization of movement can have an impact on these patients through improving their modulation by inducing the activation of movement patterns without nociceptive inputs that would lead to central desensitization.

The pressure pain threshold of the sensitivity to the painful stimulus and its modification implies the mechanisms involved. More than half of the patients who underwent the intervention presented changes beyond the MDC in the local pressure pain threshold (malleolus) and remote pressure pain threshold (extensor carpi radialis longus). These results suggest changes in peripheral and central sensitization mechanisms. As much as 41% of patients who underwent the intervention exhibited changes in the pressure pain threshold at L5, above the minimum detectable change of that variable. This therapeutic approach in patients with arthropathy can promote a decrease in neurological sensitization, facilitating a change in the somatosensory profile affected in patients with haemophilia (38).

This study reports an improvement in ankle range of motion in patients who underwent the intervention compared with the control group. However, based on the minimal detectable change, these changes and their clinical relevance should be interpreted with caution. Improved ankle mobility affects lower limb functionality in these patients (39). Accordingly, the improvements observed in range of motion together with the motor reprogramming induced by the visualization of movement may make it advisable to use immersive virtual reality as a complementary intervention in the approach to these patients.

Pain catastrophizing can increase the painful experience, leading to avoidance behaviour and favouring kinesiophobia (8). The suitability of carrying out an approach on cortical reorganization and psychosocial factors in patients with kinesiophobia has been described.

Gradual exposure to the adverse stimulus through virtual reality has been used to address other phobias such as the fear of flying or social anxiety (40). No intergroup differences were found in this study, which may be due to the fact that kinesiophobia prior to the study was, on average, below 27 points.

### Study limitations

This study presents some limitations that should be considered. The fact that patients were not blinded is an important limitation. Although the intake of analgesic drugs prior to the intervention was evaluated, the use of these drugs could not be monitored due to their self-administration without a doctor’s prescription in many cases. Their impact is a factor that must be taken into account for the interpretation of the results. The intervention was carried out on the patients’ mobile phone, with each of the various smartphone models, having respective operating systems and screen sizes. Use of a single stand-alone headset system, with greater video quality, realism, and immersion, would have been an ideal option to unify the intervention for all patients.

### Conclusion

A 180-degree immersive VR video-based visualization of movement is an effective tool for addressing the intensity of pain in patients with haemophilic ankle arthropathy. This intervention can improve conditioned pain modulation and ankle range of motion in this population. Visualization of movement can produce a clinically relevant change in the pressure pain threshold locally, at the spinal segment, and at the unrelated neurological level (central sensitization).

## Trial registration number

id NCT04549402.
